# Delayed Lactogenesis II and potential utility of antenatal milk expression in women developing late-onset preeclampsia: a case series

**DOI:** 10.1186/s12884-018-1693-5

**Published:** 2018-03-15

**Authors:** Jill Demirci, Mandy Schmella, Melissa Glasser, Lisa Bodnar, Katherine P. Himes

**Affiliations:** 1 Department of Health Promotion & Development, University of Pittsburgh School of Nursing, 440 Victoria Building, Pittsburgh, PA 15261 USA; 2 Department of Pediatrics, University of Pittsburgh School of Medicine, Pittsburgh, PA USA; 3 Department of Epidemiology, University of Pittsburgh Graduate School of Public Health, Pittsburgh, PA USA; 4 Department of Obstetrics, Gynecology and Reproductive Sciences, University of Pittsburgh School of Medicine, Pittsburgh, PA USA; 5 Magee-Womens Research Institute, Pittsburgh, USA

**Keywords:** Pre-eclampsia, Hypertension, Pregnancy-induced, Breast feeding, Lactation, Breast milk expression

## Abstract

**Background:**

Preeclampsia is a multi-system, hypertensive disorder of pregnancy that increases a woman’s risk of later-life cardiovascular disease. Breastfeeding may counteract the negative cardiovascular sequela associated with preeclampsia; however, women who develop preeclampsia may be at-risk for suboptimal breastfeeding rates. In this case series, we present three cases of late-onset preeclampsia and one case of severe gestational hypertension that illustrate a potential association between hypertensive disorders of pregnancy and suboptimal breastfeeding outcomes, including delayed onset of lactogenesis II and in-hospital formula supplementation.

**Case presentation:**

All cases were drawn from an ongoing pilot randomized controlled trial investigating the impact of antenatal milk expression versus an education control on breastfeeding outcomes. All study participants were healthy nulliparous women recruited at 34–36^6/7^ gestational weeks from a hospital-based midwife practice. The variability in clinical presentation among the four cases suggests that any effect of hypertensive disorders on breastfeeding outcomes is likely multifactorial in nature, and may include both primary (e.g., preeclampsia disease course itself) and secondary (e.g., magnesium sulfate therapy, delayed at-breast feeding due to maternal-infant separation) etiologies. We further describe the use of antenatal milk expression (AME), or milk expression and storage beginning around 37 weeks of gestation, as a potential intervention to mitigate suboptimal breastfeeding outcomes in women at risk for preeclampsia and other hypertensive disorders of pregnancy.

**Conclusions:**

Additional research is needed to address incidence, etiology, and interventions, including AME, for breastfeeding issues among a larger sample of women who develop hypertensive disorders of pregnancy.

## Background

Preeclampsia is a heterogeneous and multi-system hypertensive disorder of pregnancy that affects ≈3–5% of pregnancies globally, causing significant maternal and neonatal/infant morbidity and mortality [1, 2]. The development of preeclampsia stems from the interactions between a dysfunctional placenta and maternal constitutional factors, which culminate in systemic endothelial dysfunction (e.g., maternal hypertension, proteinuria, activation of coagulation cascade) [3–5]. Preeclampsia is diagnosed when a woman presents with new onset hypertension after 20 weeks’ gestation with proteinuria and/or signs of multi-system involvement (e.g., thrombocytopenia, renal insufficiency). Preeclampsia is characterized as early (< 34 weeks’ gestation) or late (≥ 34 weeks’ gestation) and with/without severe features indicative of multi-system organ involvement [6]. Certain pre-pregnancy and pregnancy related factors, including pre-pregnancy obesity, chronic hypertension, type 1 and 2 diabetes mellitus, advanced maternal age, and nulliparity are associated with an increased risk of preeclampsia [7].

Although the acute signs/symptoms generally resolve in the days and weeks following delivery [8], preeclampsia is associated with an increased risk for cardiovascular disease remote from pregnancy. In women with a history of preeclampsia, the risk of ischemic heart disease and cerebrovascular events is doubled, the risk of hypertension is tripled, and the risk of incident heart failure is quadrupled [9–12]. Women with hypertensive disorders of pregnancy also have a significantly higher risk of mortality related to diabetes, ischemic heart disease, and diabetes [13, 14]. Children exposed to a preeclamptic pregnancy may be at increased risk for cardiovascular dysfunction in adolescence and adulthood [15–17].

Breastfeeding may be one way to counteract the negative cardiovascular sequelae associated with preeclampsia. Increased duration of lactation in women is associated with reduced later life risk of hypertension, type II diabetes, hyperlipidemia, metabolic syndrome, subclinical and clinical cardiovascular disease, and cardiovascular mortality [18–22]. Breastfed children have a reduced risk of childhood obesity and diabetes [23, 24]. Paradoxically, however, women who develop preeclampsia appear to be at-risk for premature breastfeeding discontinuation and early formula use [25, 26], for reasons which have not been previously described.

Here, we present three cases of late-onset preeclampsia and one case of severe gestational hypertension that illustrate a potential association between hypertensive disorders of pregnancy and suboptimal breastfeeding outcomes, including delayed onset of lactogenesis II and in-hospital formula supplementation. Lactogenesis II is defined as the onset of copious milk production, which typically occurs between 48 and 72 h postpartum; onset after 72 h is considered delayed and is associated with unintended breastfeeding reduction and cessation [27, 28]. Furthermore, we describe the use of antenatal milk expression (AME), or milk expression and storage beginning around 37 weeks of gestation, as a potential intervention to increase breastfeeding success in women at risk for preeclampsia and other hypertensive disorders of pregnancy.

All cases were drawn from an ongoing pilot randomized controlled trial investigating the impact of antenatal milk expression versus an education control on breastfeeding outcomes. All trial participants were healthy nulliparous women with singleton pregnancies recruited at 34–36^6/7^ gestational weeks from a hospital-based midwife practice in the northeastern United States. Participants were recruited via study flyer and at their prenatal appointments after review of electronic medical records for eligibility criteria. At enrollment, women had no major risk factors for insufficient milk (e.g., breast hypoplasia, polycystic ovarian syndrome, diabetes) or preterm labor (e.g., vaginal bleeding after the first trimester, congenital anomalies, polyhydramnios). The AME protocol was modeled after an existing randomized controlled trial of AME in women with diabetes [29] and involved demonstration of hand-expression of milk beginning at 37 weeks of gestation with a study lactation consultant, weekly visits with the same lactation consultant to reinforce technique, and at-home, independent milk expression and collection one to two times per day for up to 10 min, for up to 40 gestational weeks. Women were provided containers in which to collect and freeze milk. Among women who brought milk to the hospital, they were advised to transport it in an insulated bag or cooler with two icepacks, and milk was subsequently stored in the hospital refrigerator for a maximum of 24 h. Women in the education group received weekly hand-outs addressing common breastfeeding problems from gestational weeks 37 to 40. We followed-up with all study participants at 1–2 weeks and 3–4 months postpartum to assess breastfeeding status and conduct interviews about experiences with AME.

Cases were obtained from the first third of participants recruited in the study, as an early trend was observed soon after enrollment commenced regarding development of hypertensive disorders of pregnancy and suboptimal breastfeeding outcomes. Case data was compiled from study participants’ electronic medical records (e.g., details on labor and delivery, feedings in hospital), study surveys (e.g., breastfeeding plans, timing of lactogenesis II, feedings after hospital discharge), antenatal breast milk expression logs (e.g., volume and timing of milk collection), and interviews (e.g., perceived impact of AME). Data were organized in tables to facilitate comparison across participants. In the presentation of all cases, we adhered to CARE case report guidelines guidelines, which specify inclusion of particular elements to increase transparency and accuracy of data (e.g., inclusion of dose/strength/duration of intervention, patient perspective).

## Case presentation

A summary of the cases can be found in Table 1.

**Table 1:** Case subject characteristics

CASES				
	1: “M”	2: “C”	3: “K”	4: “S”
STUDY DATA				
Study group assignment	AME	Education	AME	AME
Number of intervention study visits prior to delivery (max of 4)	3	0	1	0
DEMOGRAPHICS				
Age	26	34	36	35
Race & ethnicity	Non-Hispanic, White	Non-Hispanic, White	Hispanic, Other	Non-Hispanic, White
Marital status	Married	Married	Married	Married
Highest level of education	Bachelors	Some college	Bachelors	PhD
OBSTETRIC & HEALTH HISTORY				
Pre-pregnancy BMI	22.6	20.2	24.7	30.0
Gestational weight gain (lbs)	33	57	33	32
Relevant health history	Supraventricular tachycardia	Anxiety, depression (no medications)	N/A	Anxiety, depression (75 mg sertraline daily)
Gravida status	1 (previous spontaneous abortion in first trimester)	0	0	0
PREECLAMPSIA/HYPERTENSIVE DISORDER				
Classification	Preeclampsia with severe features (thrombocytopenia)	Preeclampsia without severe features	Preeclampsia without severe features	Gestational hypertension (with severe range blood pressures)
Weeks at diagnosis	39^3/7^	36^6/7^	39^0/7^	37^4/7^
Magnesium sulfate administration	56.4 g over 49 h	n/a	n/a	83.8 g over 44 h
Placenta pathology report^a^	No diagnostic abnormalities; 557 g, 75th percentile	390 g, 10th percentile; Features of maternal vascular malperfusion	No diagnostic abnormalities; 383 g, 10th percentile	No diagnostic abnormalities; 353 g, 10th percentile
LABOR & DELIVERY				
Weeks gestation at delivery	39^4/7^	37^0/7^	39^1/7^	37^5/7^
Onset of labor	Natural	Induced	Induced	Induced
Augmentation of labor	Pitocin, misoprostol, foley bulb, artificial rupture of membranes	Misoprostol	Dinoprostone, Pitocin	Pitocin, misoprostol, foley bulb, artificial rupture of membranes
Labor anesthesia	Epidural	Spinal, general	Epidural, spinal	Epidural
Type of delivery (and indication)	Cesarean section (arrested dilation and worsening preeclampsia)	Cesarean section (fetal intolerance)	Cesarean section (fetal intolerance)	Vaginal
Duration of labor in hospital (hours)	36	15	25	24
Duration of second stage of labor (hours)	N/A	N/A	N/A	0.5
Total intravenous fluids during labor and postpartum (mL)	8148 over 85 h	6620 over 81 h	3408 over 7 h	6816 over 41 h
POSTPARTUM				
Maternal disposition after birth	ICU	Mother-baby unit	Mother-baby unit	Mother-baby unit
Infant disposition after birth	NICU	Well-baby nursery	Well-baby nursery	Well-baby nursery
Infant birthweight (g)	3766	2927	2947	2580
Infant weight change from birthweight at hospital discharge	−7.6%	− 6.6%	− 6.1%	− 6.2%
Infant Apgar scores 1 & 5 mins	2, 7	8, 9	7, 9	6, 8
Maternal hospital LOS (days)	4	5	4	2
Infant hospital LOS (days)	3	4	3	2
BREASTFEEDING				
Lactation consult(s) in hospital	No	Yes (×4)	Yes (×1)	Yes (×2)
Total volume of formula in hospital (mL)	0	455	0	199
First at-breast feed in hospital after delivery (hours)	1.5	3.5	1.3	1.5
First use of formula in hospital after delivery (hours)	N/A	1.5	N/A	15.5
Milk expression in hospital (e.g., hand expression or pump)	Yes	Yes	Yes	Yes
Maternal perception of onset of lactogenesis II	Day 5	Day 5	Day 2	Day 4
Breastfeeding status at 1–2 weeks^b^	Exclusive breastfeeding	Exclusive breastfeeding	Exclusive breastfeeding	Medium partial breastfeeding
Breastfeeding status at 3–4 months^b^	Exclusive breastfeeding	High partial breastfeeding	Exclusive breastfeeding	Exclusive breastfeeding

^a^Percentiles are placental weights-for-gestation

LOS = length of stay

^b^Breastfeeding status classifications based upon definitions provided by Labbok and Krasovec, where exclusive breastfeeding equates with 100% of all feeds as breast milk, high partial breastfeeding is 80% or more of all feeds as breast milk, and medium partial breastfeeding is 20–80% of all feeds as breast milk

Labbok M, Krasovec K. Toward consistency in breastfeeding definitions. *Studies in Family Planning.* 1990;21(4):226–230

### Case 1: M

M was a healthcare professional with some formal lactation training. During pregnancy, she experienced normal, physiologic changes to her breasts, including breast growth and soreness, darkening of the areola, and leaking milk. After delivery, M planned to exclusively breastfeed for six months and continue any breastfeeding to one year postpartum.

Assigned to the AME study intervention, M expressed milk at least daily at home from 37 weeks of gestation to delivery (14 days, 15 total expression sessions, 60 mL total milk volume). All expressed milk was stored in her home freezer, and approximately half of stored milk was brought to the hospital by her partner on Day 2 after delivery. M experienced no problems with AME, such as physical discomfort or issues related to privacy, convenience, or milk transport to/use in the hospital.

At 39^3/7^ weeks’ gestation, M presented to labor and delivery triage with painful contractions every five minutes. Her blood pressure was also elevated (reaching 140 s/110 s mmHg) and labs revealed thrombocytopenia (platelet count = 94,000/μl). Protein/creatinine (p/c) ratio was equivocal at 0.17. M was diagnosed with preeclampsia with severe features and intravenous magnesium sulfate treatment was initiated according to protocol. An epidural was placed approximately 17 h into labor.

Despite augmentation of labor, including up-titration of oxytocin (Pitocin, Par Pharmaceutical Inc., Spring Valley, New York) and artificial rupture of membranes, 24 h into labor there was minimal cervical change and worsening preeclampsia-related laboratory values (e.g., liver and renal function labs trending upward, decreased urine output). This prompted a decision to proceed with a cesarean section, which was completed without maternal complication. At delivery, M’s infant (female) was apneic and bradycardic, requiring bag and mask resuscitation. Resuscitation efforts were successful, and the infant was transferred to the neonatal intensive care unit (NICU) with continuous positive airway pressure (CPAP) for further monitoring. In the NICU, the infant was weaned to room air, and after one hour, was transferred to the well-baby nursery. Postoperatively, M was transferred to the intensive care unit (ICU) for further monitoring and magnesium sulfate continuation for approximately 24 h. On postpartum day two, M was transferred to the mother-baby unit with improving preeclampsia-related laboratory values.

The first feed, which was at-breast, occurred about 1.5 h after delivery in the ICU. M reported the infant latched on “decently” (no LATCH score available; note that LATCH scores are used in the clinical context to document success of individual breastfeeding sessions according to five criteria; scores range from 0 to 10, with a higher score indicative of a more successful breastfeeding session [30]). During M’s ICU stay, she alternated between at-breast feeds and bottle-feeding fresh colostrum that she hand-expressed or pumped. M breastfed exclusively the rest of hospitalization with LATCH scores of 9–10 and supplemented once overnight with a bottle of breast milk she expressed antenatally (25 mL), due to fatigue. She did not have a lactation consult in the hospital.

Upon discharge at three days, M did not perceive an increase in milk supply, and that night she supplemented with an additional 25 mL of the antenatally expressed milk due to her concern about lack of wet diapers. At M’s follow-up interview at seven days postpartum, she noted that her milk did not fully “come in” until Day 5.

During the interview, M was enthusiastic about her experience with AME; she attributed a progressively increasing volume of milk expressed and leaking during pregnancy to AME, which increased her breastfeeding confidence. She also noted that AME created a “back-up” milk supply and seemed to increase the availability of colostrum in the postpartum period before her milk came in. M credited AME with avoiding formula supplementation after birth, within contexts that typically result in unintended formula supplementation (e.g., exhaustion with prolonged labor, mother-infant separation, impaired mental clarity with magnesium sulfate administration). In her interview she stated, “I pumped that night in the ICU and was able to get 8cc’s out and give that to [my baby] so I could rest. And I thoroughly believe that would never have happened had I not been doing the study and AME, because just of what I’ve seen in the hospital, the first time you pump, you get like drops.” At the 3–4 month follow-up interview, M reported continued exclusive breastfeeding.

### Case 2: C

C was randomized to the education only study group. She planned to exclusively breastfeed for 6 months and continue any breastfeeding up to 1 year. During pregnancy, she experienced breast growth and soreness.

C presented to labor and delivery triage at 36^6/7^ weeks gestation after a routine obstetrical visit exam revealed elevated blood pressure of 138/94 mmHg and 1+ protein on a urine dipstick. Upon exam in triage, C’s blood pressure rose to 140 s/90s, and she had an elevated urine p/c ratio of 0.98. She was diagnosed with preeclampsia without severe features, and induction of labor commenced. Approximately 8 hours into the induction, repetitive late decelerations in fetal heart rate were noted, and the decision was made to proceed with a cesarean section. After delivery of a healthy male infant, C developed uterine atony and carboprost and misoprostol were administered, along with a Pitocin drip and a uterine B-Lynch suture. Due to waning effect of spinal anesthesia, C was placed under general anesthesia during operative closure. After recovery, C and her infant were transferred to the mother-baby unit.

C’s postoperative course was complicated by general malaise, persistent nausea and vomiting, and tachycardia. The latter two issues were attributed to postoperative ileus and under-resuscitation after significant blood loss at delivery. While C was in the operating room 1.5 h after delivery, her partner fed the infant a bottle of formula (undocumented volume). Approximately 3.5 h after delivery, C fed her infant at-breast for the first time, with an 8/10 LATCH score. For the next 40 h, C fed her infant at-breast with LATCH scores of 7–9, with the exception of 30 mL of formula overnight on the second night in the hospital due to infant hypoglycemia (undocumented if symptomatic). Early the following morning and for the next 24 h, the infant was fed only bottles of formula; per maternal self-report at the study hospital visit, this was due to her malaise. During this period, C was provided with a breast pump, and on days 3 to 4 after delivery, the infant then received a combination of at-breast feeds and formula or breast milk via bottle. C received daily lactation consults during her hospitalization, beginning 34 h after delivery. During consults, basic breastfeeding education was provided, including anticipatory guidance about potential issues feeding an early term infant, as well as assistance at-breast.

C’s symptoms gradually improved, and she was discharged on post-operative Day 4. She remained normotensive during the postpartum period. At study follow-up at 12 days postpartum, C reported that she felt her milk “came in” on postpartum Day 5, and she was exclusively breastfeeding at-breast. At the 3–4 month follow-up, C was breastfeeding at-breast for most feeds. She estimated that formula made up about 10% of her infant’s total feeds in a 24 h period.

### Case 3: K

K planned to breastfeed exclusively for 6 months and continue any breastfeeding for 12 months postpartum. During pregnancy, she experienced breast growth, soreness, and darkening of areola. K was randomized to receive AME but completed only the first study visit with AME instruction and 5 days of AME at home (seven milk expression sessions) prior to delivery. During milk expression sessions, several drops of milk were expressed, but not saved. K reported no problems with AME, with the exception of minor physical discomfort during expression related to breast soreness in pregnancy and some concern that perceived lack of milk flow in pregnancy might be predictive of impaired milk supply postpartum.

K presented to labor and delivery triage after a growth ultrasound at 39^0/7^ weeks gestation, which revealed fetal tachycardia, a non-reactive non-stress test and elevated maternal blood pressure. Upon arrival in triage, K was found to have new-onset hypertension in the 140 s–150 s/90s–100 s and a p/c ratio of 1.03. She was diagnosed with preeclampsia without severe features. K was admitted for an induction of labor, and cervical ripening proceeded with dinoprostone placement. As labor progressed, platelets began to trend down slightly with a low of 114,000/μL approximately 1 hour prior to delivery. Approximately 17 h into the induction, a Pitocin drip was begun and K received an epidural. At 20 h after the induction commenced, fetal heart monitoring revealed reduced variability, tachycardia, and recurrent late decelerations. Intrauterine resuscitation efforts (e.g., maternal repositioning, oxygen) did not improve the tracing, and when a vaginal exam was attempted, spontaneous rupture of membranes occurred with thick meconium-stained fluid. Given concern for fetal status and limited labor progress, the obstetric team proceeded with a cesarean section and delivered a healthy male infant. During the closure, K experienced mild uterine atony requiring uterotonic agents (carboprost, Pitocin, misoprostil). Both K and infant were transferred to the well mother-baby unit after recovery, with normotensive maternal blood pressure readings.

K breastfed her infant for the first time approximately 1.3 h after delivery, with a LATCH score of 6/10. She continued to exclusively breastfeed throughout the 3-day postpartum hospitalization, with LATCH scores ranging from 6 to 9/10. A lactation consult occurred at 48 h post-delivery. The lactation consultant note indicated that K was breastfeeding well and hand-expressing milk easily. “Pointers” were provided on latch and positioning.

At study follow-up at 12 days postpartum, K reported that her milk “came in” at 2 days postpartum and that she had no breastfeeding concerns. She was exclusively breastfeeding, and felt that AME had increased her confidence in her ability to produce milk after birth and allowed her to “practice” handling breasts and the mechanics of breastfeeding prior to birth. She also attributed AME to her milk “coming in sooner” after birth. At 3–4 months postpartum, K was exclusively breastfeeding at-breast.

### Case 4: S

S experienced normal, physiologic changes to her breasts during pregnancy, including growth, areola darkening, increased breast vascularization, and leaking milk. S planned to breastfeed without formula in the postpartum period for as long as possible. Although randomized to AME, S’s exam at her obstetric visit at 37^4/7^ weeks gestation revealed new-onset hypertension in the range of 130 s/90s mmHg, and she did not receive the intervention.

Upon admission to triage for further evaluation, her blood pressure increased to 160 s/110 s, with no other signs or symptoms of preeclampsia. She received a single dose of labetalol 20 mg IV. Induction of labor then commenced, and S received a 2 g/h magnesium sulfate drip for seizure prophylaxis. A Pitocin drip was begun approximately 6 h into labor, and membranes were artificially ruptured 10 h later. S received an epidural approximately 20 h into labor. Shortly thereafter, S delivered a female infant who required no resuscitation. S and her infant remained together throughout the recovery period and were later transferred to the well mother-baby unit. S continued to receive magnesium sulfate for approximately 24 h post-delivery, and her blood pressures returned to the 120 s/70s mmHg.

S first attempted at-breast feeding 1.5 h after delivery, with a LATCH score of 6/10. In the ensuing hours, nursing notes indicated no LATCH scores above 6 and several unobserved breastfeeding sessions where no sustained latch was achieved. S first supplemented with formula during the night following her morning delivery (22 mL), after an unsuccessful breastfeeding attempt. Formula was continued in lieu of breastfeeding the remainder of the night, with attempts to breastfeed the following morning followed by formula supplementation. A similar pattern was followed during the remainder of the hospitalization. S saw a hospital lactation consultant approximately 24 h after delivery who provided basic breastfeeding education, including feeding cues and instructions for pumping after unsuccessful breastfeeding attempts. She returned 5 hours later to observe a feed and demonstrate hand-expression of colostrum and the use of a nipple shield. The lactation consultant returned the following morning, and noted that S reported the infant “‘frustrated and screaming [during breastfeeding].’ [Mother] then gives a bottle [of formula] and pumps.”

At study follow-up at 11 days of life, S reported feeling her milk “come in” at approximately 4 days postpartum. After hospital discharge, she had continued to feed a combination of breast milk (at-breast and via bottle) and formula, with approximately a third of feeds in the past 24 h being breast milk. She continued to use the nipple shield and had received breastfeeding help from family and friends. She continued to pump after formula feeding, roughly six times in a 24 h period, with about 4 oz. of breast milk expressed and fed to the infant. She attributed milk supply issues to a five-day course of furosemide (Lasix, Sanofi Aventis, Bridgewater, New Jersey) she was prescribed 5 days after delivery for peripheral edema and continued elevated blood pressures (“It has to be the Lasix. It’s drying you up everywhere.”). At 3–4 months postpartum, S reported exclusive breast milk feedings, with the majority of feedings as expressed milk via bottle. She continued to use the nipple shield for at-breast feedings.

## Discussion and conclusions

Three of the cases presented here illustrate a potential relationship between late-onset preeclampsia and other hypertensive disorders of pregnancy and early breastfeeding problems, including delayed onset of lactogenesis II and in-hospital formula use. Case 3 represents an exception to this association, with normal timing of lactogenesis II and exclusive breastfeeding in-hospital. The variability in clinical presentation among the four cases supports that any potential effect of preeclampsia or other hypertensive disorders of pregnancy on breastfeeding outcomes is likely multifactorial in nature, with possible primary etiology (e.g., preeclampsia disease course itself) and secondary causal factors (e.g., underlying maternal issue contributing to both preeclampsia and lactation problems, preeclampsia treatments).

We found two studies addressing the relationship between hypertensive disorders of pregnancy and breastfeeding outcomes, but none examining timing of lactogenesis II. Cordero et al. [25] found in their retrospective medical record analysis of 281 women diagnosed with severe preeclampsia and delivering ≥ 34 weeks of gestation, 51% of the overall sample and 78% of those intending to breastfeed initiated breastfeeding; however, only 33% of the sample was exclusively breastfeeding and 22% receiving any breast milk at the time of hospital discharge, which was lower than U.S. averages at the time but perhaps comparable to the region in which the study occurred [31, 32]. In a retrospective cohort study involving mothers with hypertensive disorders of pregnancy and matched controls, breastfeeding intention and continuation at 1 month postpartum was significantly lower among women with preeclampsia than control women [26]. These studies lend credence to a potential negative effect of preeclampsia or related factors on breastfeeding, though mechanism of action is unclear.

Three of four cases presented with lactogenesis II occurring after 3 days postpartum. Onset of lactogenesis II is triggered by a complex hormonal cascade, initiated by delivery of the placenta and a concomitant precipitous drop in progesterone. The methods by which various factors facilitate or impede this process are not well understood. Delays in lactogenesis occur at a rate of 23–44% in the U.S. [28, 33]. This is clinically significant, as delayed lactogenesis, and its corollary—formula supplementation, are both are strong prognosticators of early, unintended breastfeeding cessation [28, 34]. Delayed lactogenesis is more likely to occur among primiparous, obese, and diabetic women, as well as those who have a prolonged second stage of labor (greater than 2–3 h for nulliparous women) [35], stressful labor or delivery, cesarean section, higher birthweight infant, postpartum edema, or a perception of ineffective breastfeeding during the first 24 h postpartum [33, 36–39]. Women in the presented cases possessed a number of these risk factors, many of which are also associated with preeclampsia and/or its treatment, including primipara status, delivery by cesarean section, stressful/prolonged labor and delivery, and early breastfeeding problems, including formula use.

While postpartum edema was not tracked as a potential cause of delayed lactogenesis II, it is a common occurrence in preeclampsia as vasculature becomes “leaky,” resulting in third-spacing of fluids [40]. It is thus possible that lower intravascular volume contributed to poor diffusion of water into mammary alveoli and delayed the onset of copious milk production until fluid balance was restored. Alternately, postpartum breast edema can contribute to difficulty in early breast milk removal, a risk factor for delayed lactogenesis II [41].

Other factors related to hypertensive disorders of pregnancy may contribute to an unfavorable endocrine profile for establishment of lactation, including stress [38, 42] and obesity [43, 44], the latter potentially relevant for Case 4 with a BMI of 30. Placental dysfunction, which typifies preeclampsia, has been generically noted as a potential contributor to delayed onset of lactation [45]; it is theoretically possible that placental hormones impacting proliferation of glandular breast tissue in preparation for breastfeeding (e.g., progesterone) are compromised in preeclampsia.

Postpartum hemorrhage, as occurred for Case 2, has the potential to contribute to delayed or insufficient milk and negative breastfeeding outcomes through several mechanisms. These include anemia and necrotic damage or shock to the pituitary gland from hypovolemia. Pituitary dysfunction affects secretion of key lactation hormones, including prolactin necessary for milk production [46]. Labor complications associated with preeclampsia, including inductions, cesarean sections, and prolonged labors are also risk factors for postpartum hemorrhage.

It is possible that medications used to treat and manage preeclampsia impacted milk supply in these cases. For example, diuretics such as Case 4 received, may contribute to a fluid deficit in relation to the volume needed for adequate milk production [47]. Case 4 was also taking a selective serotonin reuptake inhibitor (SSRI) daily; SSRIs are associated with delayed lactogenesis, potentially through perturbation of the mammary gland epithelial cells [48]. Magnesium sulfate therapy is routinely utilized for seizure prophylaxis in women with preeclampsia during the intrapartum and postpartum periods. A case report from 1993 describes a woman who received ≈ 4 days of magnesium sulfate therapy (102 g total) for management of preeclampsia and experienced delayed lactogenesis to postpartum day 10 [49]. Traditional reasons for delayed lactogenesis (e.g., delayed at-breast feeding, decreased breast stimulation) were ruled out, and it was hypothesized that the disease process or the magnesium therapy itself impacted lactogenesis. Other studies have found a dose-dependent association of magnesium sulfate therapy with delayed initiation of breastfeeding [50] and factors that impact delayed breastfeeding, including decreased infant suckling behavior, lower Apgar scores, hypotonia, and NICU admissions [51, 52]. Two of the four cases presented in this case series received magnesium sulfate therapy (Case 1: 56.4 g total; Case 4: 83.8 g total) and experienced delayed lactogenesis II. Unfortunately, research investigating the impact of magnesium sulfate therapy on lactogenesis is still lacking.

Reduced opportunity for early and effective breast stimulation and emptying, as may occur with maternal infant separation and medical complications in preeclampsia management, are known risk factors for reduced breast milk production [33, 53]. Research suggests that the earlier postnatal milk removal and suckling occurs, the more rapid the conversion to lactogenesis II (copious mature milk) post-birth and the larger the postpartum milk supply [54, 55]. One explanation is the prolactin receptor theory, which posits that early breast stimulation increases the number of prolactin receptors in breast tissue; these receptors set the theoretical maximum volume of milk production and may only be amenable within a short period around birth [56]. This may be particularly relevant for Cases 2 and 4 who delivered prior to 39 weeks gestation, as preterm and early term infants generate reduced negative pressure during suckling and removal of less milk [57].

As illustrated in Cases 1 and 3, antenatal milk expression (AME) may address a number of breastfeeding complications experienced by women with late onset preeclampsia. For example, milk stored in pregnancy can be used as supplemental feeding when at-breast feeds are not possible or successful following a complicated birth. AME may also increase prenatal confidence and commitment to breastfeeding, allowing mothers to persevere despite early postpartum setbacks (however, AME may also have unintended consequences of decreasing confidence if mothers are not able to express any milk or only small volumes). Finally, existing research [58, 59] and anecdotal feedback in our study suggest that AME may contribute to increased volume of milk both prior to and following lactogenesis II and improved short-term breastfeeding outcomes. Forster et al. [58] found that AME beginning at 36 weeks of gestation among diabetic women at low-risk for birth complications did not increase risk of NICU admission or rate of preterm birth. Because nipple stimulation in pregnancy can theoretically lead to uterine contractions and perhaps placental perfusion problems, the authors advised against unsupervised AME implementation in other high-risk groups. In our study, AME was not continued after women were diagnosed with any pregnancy complications. More research is warranted to understand the risks and benefits of AME among women who develop preeclampsia and other issues associated with postnatal breastfeeding problems. In the interim, women with prenatal *risk factors* for these problems, including nulliparity, with an otherwise low-risk pregnancy, may benefit from AME prior to onset of disease.

In this sample of women with hypertensive disorders of pregnancy, despite early setbacks and formula use, all were breastfeeding with little to no formula use by three to 4 months postpartum. It is unclear whether this would have been the case in a more diverse sample of women with less breastfeeding education and support. Thus, limitations of this analysis include the generalizability of findings based on the case study format and the demographically homogenous sample of women. Strengths include utilization of multiple data sources enabling methodological triangulation (e.g., electronic medical record data and maternal self-report of in-hospital feedings) and the prospective nature of data collection from pregnancy through the postpartum period, minimizing the potential for recall bias. Future research should address incidence, etiology, and interventions for breastfeeding issues among larger samples of women who develop late onset preeclampsia, while controlling for confounding variables.

## Abbreviations

AME: Antenatal milk expression
CPAP: Continuous positive airway pressure
ICU: Intensive care unit
LATCH: breastfeeding scoring system based on five criteria represented by the LATCH acronym: L: latching of infant onto breast; A: audible swallows; T: type of nipple; C: comfort of the mother during breastfeeding; H-hold/position used during breastfeeding and assistance required
NICU: Neonatal intensive care unit
p/c ratio: Protein/creatinine ratio
SSRI: Selective serotonin reuptake inhibitor
